# TRPM8 channel as a novel molecular target in androgen-regulated prostate cancer cells

**DOI:** 10.18632/oncotarget.3948

**Published:** 2015-04-29

**Authors:** Swapna Asuthkar, Kiran Kumar Velpula, Pia A. Elustondo, Lusine Demirkhanyan, Eleonora Zakharian

**Affiliations:** ^1^ University of Illinois College of Medicine, Department of Cancer Biology and Pharmacology, Peoria, IL, USA; ^2^ Dalhousie University, Halifax, NS, Canada

**Keywords:** prostate cancer (PC), transient receptor potential melastatin 8(TRPM8) ion channel, testosterone, DHT (5α-dihydrotestosterone), androgen receptor (AR)

## Abstract

The cold and menthol receptor TRPM8 is highly expressed in prostate and prostate cancer (PC). Recently, we identified that TRPM8 is as an ionotropic testosterone receptor. The TRPM8 mRNA is expressed in early prostate tumors with high androgen levels, while anti-androgen therapy greatly reduces its expression. Here, from the chromatin-immunoprecipitation (ChIP) analysis, we found that an androgen response element (ARE) mediates androgen regulation of *trpm8*. Furthermore, using immunofluorescence, calcium-imaging and planar lipid bilayers, we identified that TRPM8 channel is functionally regulated by androgens in the prostate. Although TRPM8 mRNA is expressed at high levels, we found that the TRPM8 protein undergoes ubiquitination and degradation in PC cells. The mass-spectrometry analysis of TRPM8, immunoprecipitated from LNCaP cells identified ubiquitin-like modifier-activating enzyme 1 (UBA1). PYR-41, a potent inhibitor of initial enzyme in the ubiquitination cascade, UBA1, increased TRPM8 activity on the plasma membrane (PM) of LNCaP cells. Furthermore, PYR-41-mediated _PM_TRPM8 activity was accompanied by enhanced activation of p53 and Caspase-9. Interestingly, we found that the *trpm8* promoter possesses putative binding sites for p53 and that the overexpression of p53 increased the TRPM8 mRNA levels. In addition to the genomic regulation of TRPM8 by AR and p53, our findings indicate that the testosterone-induced _PM_TRPM8 activity elicits Ca^2+^ uptake, subsequently causing apoptotic cell death. These findings support the strategy of rescuing _PM_TRPM8 expression as a new therapeutic application through the regulation of PC cell growth and proliferation.

## INTRODUCTION

PC is one of the most common malignancies and the second leading cause of cancer-related death in American men [1]. In the early stages, the development and maintenance of PC depends on androgens, which mediates its response by binding to androgen receptor (AR) [2]. The majority of PC is androgen-dependent and responds to androgen-deprivation therapies, which consists of reducing endogenous androgen levels or directly blocking AR activity, thus causing massive apoptotic cell death [3, 4]. Unfortunately, some cancer cells escape these therapies and relapse with androgen-independent PC that are incurable [5, 6]. This shift from an androgen-responsive characteristic towards a hormone-independent tumor growth is not well understood [7, 8]. Moreover, the AR remains transcriptionally active and contributes to their androgen-independent growth [9]. Clearly, there is a clinical need to identify a target with anti-tumor activity effective both, during anti-androgen therapy and anti-androgen resistance.

The mRNA encoding TRPM8 (transient receptor potential melastatin 8) is expressed in prostate tissues, and is upregulated in early prostate tumors [10]. The loss of TRPM8 mRNA is observed during transition to androgen independence and in patients subjected to preoperative anti-androgen therapy. This suggested that TRPM8 is androgen regulated, and its loss may be associated with more advanced disease [11]. When compared with other potential PC markers (PSA, hK2 and PSCA), TRPM8 mRNA expression was shown specific for organ confined PC tumors [12]. Furthermore, retrospective studies showed variable levels of TRPM8 mRNA expression in both tumorigenic and normal prostate tissues [13]. Hence, no clear correlation of TRPM8 expression with the severity of PC was indicated.

TRPM8 is a cation channel with relatively high selectivity for Ca^2+^. TRPM8 plays a role of the cold and menthol receptor and also an important mediator of pain stimuli in the peripheral nervous system [14-17]. While the TRPM8 channel function is well characterized in the sensory neurons, the channel was initially detected and cloned from human prostate cells, where its role was unknown. Recently, we demonstrated that TRPM8 is an ionotropic testosterone receptor [18, 19]. These findings indicated that androgens regulate TRPM8 at both genomic and nongenomic levels. Although, TRPM8 expression in prostate is androgen and AR-dependent, growing evidence argues for androgen-independent effects [20, 21], which suggest that TRPM8 expression in these cells may have tumor suppressor functions. Overall, our study highlights the importance of crosstalk between androgen-TRPM8-AR regulatory loop as it relates to cancer progression and cell proliferation. We show the functional role of androgen and AR in regulating TRPM8 expression, as well as their interaction with TRPM8, thereby facilitating TRPM8 stabilization or its targeting to degradation.

## RESULTS

### The trpm8 is an androgen responsive gene

**Figure 1:**
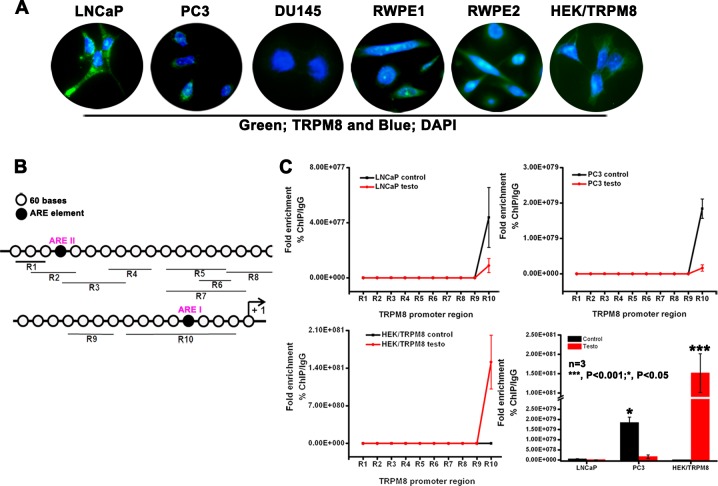
Role of ARE elements in the regulation of trpm8 gene expression **A.** Immunocytochemistry analysis for TRPM8 expression. **B.** Analysis of 2064 bp 5′-flanking region of human *trpm8* gene (ref|NW_004929306.1) identified 2 putative ARE I and ARE II sites. Quantitative PCR amplicons covering R1-R10 are indicated by black lines below. **C.** To investigate whether androgen-AR binding to the *trpm8* promoter is localized around these ARE sites, we used ChIP DNA immunoprecipitated by anti-DHT/testosterone and anti-IgG antibody. for semi-quantitative RT-PCR analysis. The ChIP specific primers covering *trpm8* promoter regions R1-R10 are listed in Stable 1. The fold enrichment of androgen interaction with different regions (R1-R10) in the *trpm8* promoter was quantified (Ct value of Input DNA/Ct value of androgen ChIP DNA) and represented graphically. The negative control anti-IgG ChIP DNA did not show amplification with R1-R10 primers.

Since TRPM8 mRNA is upregulated in PC cells, we performed immunocytochemistry analysis in different PC (LNCaP, DU145 and PC3) and prostate epithelial (RWPE1 and RWPE2) cell lines. We found that the levels of TRPM8 were abundant in LNCaP, RWPE1 and RWPE2 cells, when compared to androgen-unresponsive DU145 and PC3 cells. HEK-293 cells stably expressing TRPM8 (HEK-TRPM8) were used as a control throughout our study (Figure 1A).

TRPM8 expression is regulated by androgens [11]. In this study we investigated if the *trpm8* gene is regulated by androgens, which depends on actions of androgens to bind AR and activate it. The androgen independent pathways do not require androgens, but can be activated by growth factors acting through kinase pathways, such as the MAPK pathway or the PI3K pathway, which phosphorylate and activate AR in the absence of androgens [22]. Our aim was to study the androgen-dependent regulation of *trpm8* and several putative ARE have been indicated at the 5′ flank region of *trpm8* gene [20, 21]. To investigate whether androgen-AR complex binding to the *trpm8* promoter is localized around these ARE sites, we performed chromatin anti-DHT/testosterone immunoprecipitation (ChIP) using DNA isolated from LNCaP, PC3 and HEK-TRPM8 control, and testosterone-induced cells which were then cloned, sequenced and analyzed. The ChIP analysis identified a number of short individual DNA fragments (Supplementary Figure 1A), consisting of sequences lying between putative ARE I and II elements in the *trpm8* gene promoter (Figure 1B). To further confirm the androgen binding to ARE I and II elements, we used ChIP DNA immunoprecipitated by anti-DHT/testosterone and anti-IgG antibodies. The semi-quantitative RT-PCR was carried out using primers for regions (R) named 1–10 by scanning the first 2064 bp 5′-flanking region of the human *trpm8* gene (NW_004929306.1) identified by ChIP analysis (Figure 1B). Androgen enrichment at R10, which includes putative ARE I site, was greater than at other regions containing ARE II (R2, R3) or no ARE (R4, R5, R6, R7, R8 and R9) sites (Figure 1B and 1C). The coefficient of androgen interaction indicated that androgens/AR bind to *trpm8* promoter in a region detected by R10 primers (Figure 1C). Interestingly, when compared to testosterone-induced cells, LNCaP and PC3 control cells showed increased androgen enrichment on the *trpm8* promoter. These contradictory observations in the androgen-unresponsive PC3 cells may be due to the relatively low but detectable levels of AR mRNA [23, 24]. Whereas in HEK-TRPM8, testosterone-induced cells showed prominent androgen/AR binding of the *trpm8* promoter when compared to control cells (Figure 1C). Although, we did not detect the AR protein in PC3 cells, we observed the AR expression in HEK-293 cells by immunoblot analysis (Supplementary Figure 1B). Furthermore, these results demonstrated inverse correlation of androgen-mediated *trpm8* promoter regulation with androgen response of cells (LNCaP < PC3 < HEK-TRPM8).

### Role of androgens in TRPM8-mediated Ca^2+^ uptake

**Figure 2:**
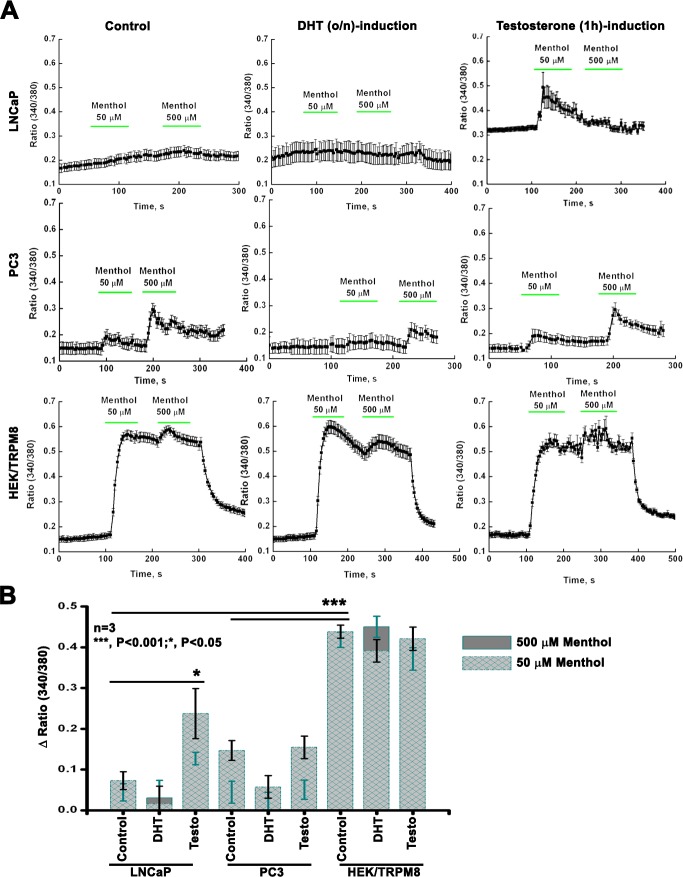
TRPM8 activity by intracellular Ca^2+^-measurements **A.** Fluorescence measurements of intracellular Ca^2+^ concentration were performed on PC cells upon DHT (o/n) and testosterone (3 h) induction by calcium-imaging. **B.** The summary of 50 μM and 500 μM menthol-induced TRPM8 responses are represented graphically.

Previous studies showed that TRPM8 acts as a Ca^2+^-permeable channel in androgen-responsive LNCaP cells [21]. To test whether androgen regulates TRPM8-mediated Ca^2+^ uptake, LNCaP, PC3 and HEK-TRPM8 control, 1 μM - DHT (o/n) and testosterone (3 h) -induced cells were analyzed using Ca^2+^ imaging (Figure 2A and 2B). The time- and dose-dependent effects of androgens were standardized initially to induce the highest TRPM8 protein expression. The standardization of conditions for TRPM8 activation was done using HEK-TRPM8 cells as described previously [25]. In these experiments TRPM8 was activated using its agonist, menthol, and resulted Ca^2+^-uptake was compared among the cell lines (Figure 2A). We found that menthol did not induce any noticeable Ca^2+^ uptake in LNCaP control or DHT-induced cells. However, testosterone-induced LNCaP cells demonstrated elevated basal Ca^2+^ levels and also responded to 50 μM menthol (Figure 2A), indicating enhanced TRPM8 activity induced by testosterone. PC3 cells showed small menthol-induced TRPM8 responses (Figure 2A and 2B).

### Role of androgens, AR and TRPM8 in PC cell viability and proliferation

**Figure 3:**
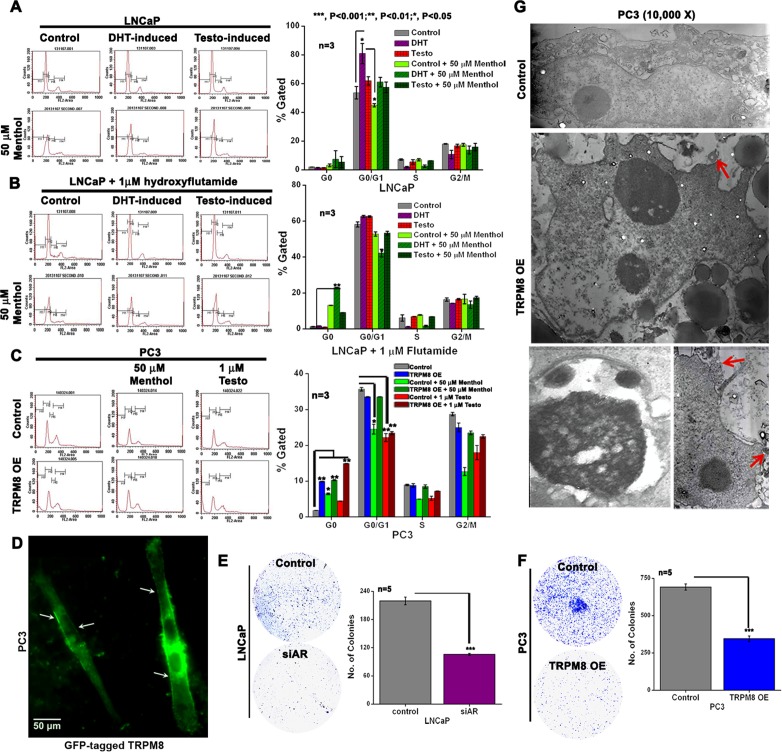
TRPM8 expression suppresses cell viability through the regulation of AR in PC cells **A.**-**B.** FACS analysis of cell cycle progression in DHT and testosterone-induced LNCaP cells both in the **A.** absence and **B.** presence of hydroxyflutamide (HF). The cells were then treated with menthol for 30 min and re-analyzed by FACS. **C.** The cell cycle progression in control and TRPM8OE PC3 cells. The cells were then treated with menthol/testosterone for 30 min and re-analyzed by FACS. The percentages of cells in sub-G1 (M1), G0/G1 (M2), S (M3) and G2/M (M4) phases of cell cycle were calculated using CellQuest Pro software and graphically represented. **D.** The PC3 cells transiently transfected with GFP-tagged TRPM8 **E.**-**F.** Clonogenic assay for **E.** LNCaP and **F.** PC3 cells. The number of colonies was quantified as the measure of clonogenicity. **G.** Transmission electron micrographs of nucleus in control and TRPM8 overexpressing PC3 cells. Images are representatives of three experiments (*n* = 3). TRPM8 overexpressing PC3 cell show blebbing of nuclear membrane (depicted by *arro*ws).

To demonstrate the role of androgens in the cell cycle progression of PC cells, FACS analysis was done in control and androgen-induced LNCaP cells. When compared to control and testosterone-induced (3 h) cells, the DHT-induced (o/n) cells demonstrated 47 % increase in G0/G1 phase. Increase in G0/G1 phase in DHT-induced LNCaP cells, could be due to many possible reasons and one among them is the induction of AR expression (Supplementary Figure 2A). Furthermore, we also investigated the effect of menthol on the cell cycle of control, DHT- and testosterone-induced LNCaP cells. Menthol, an agonist of TRPM8, is used in topical therapeutic preparations [26]. It also exerts cytotoxic activity against several cancer cell types [27], including PC cells [28]. Incubation of PC cells with 50 μM menthol for 30 min showed a decrease in G0/G1 phase by 19 % and 25 % in control and DHT-induced cells, respectively indicating the anti-proliferative role of TRPM8 on cells overexpressing AR protein (Figure 3A). Further, co-treatment with AR-antagonist, HF, abrogated the increase in DHT-induced G0/G1 phase in LNCaP cells (Figure 3B). Interestingly, the HF-treated and DHT-induced cells showed apoptotic sub-G1 phase with menthol incubation (Figure 3B). Although several studies have documented that in the androgen-deprived conditions, HF in LNCaP cells with mutant AR demonstrates an agonist activity. However, in the presence of androgens, HF acts as an antagonist and inhibits the androgen-mediated transcriptional activity [29]. Moreover, in PC3 cells, we observed that TRPM8 overexpression (TRPM8OE) leads to induction of apoptotic cell death. Further, incubation of PC3 cells with 50 μM menthol and 1 μM testosterone alone and in combination with TRPM8OE, profoundly decreased cell proliferation as evidenced by cells in the apoptotic phase increased gradually to 15-18 % (Figure 3C). We hypothesize that such an anti-proliferative effect in PC3 cells, with lower AR levels [23], could be due to the TRPM8 activity on the PM (Figure 3D). It is also important to note here that the transfection efficiency of GFP-tagged TRPM8 in PC3 cells was higher than in LNCaP (Supplementary Figure 3).

Determination of cell survival by clonogenic assay was performed in LNCaP and PC3 cells with siRNA-directed knockdown of AR (siAR) (Figure 3E) and TRPM8OE (Figure 3F), respectively. Compared with control cells, both the siAR and TRPM8OE PC cells revealed a significant *(P < 0.001)* decrease in the colony-forming efficiency. Alternatively, the electron microscopic analysis revealed formation of apoptotic bodies (chromatin condensation) with cytoplasmic vacuolization accompanied by blebbing of nuclear membranes (arrows) in TRPM8OE cells when compared to control PC3 cells (Figure 3G). These results indicate that TRPM8OE in PC cells exerts anti-proliferative effect, suggesting the importance of the TRPM8 channel as a tumor regulator.

### TRPM8 is targeted for E1 ubiquitin-activating enzyme (UBA1)-mediated ubiquitination

**Figure 4:**
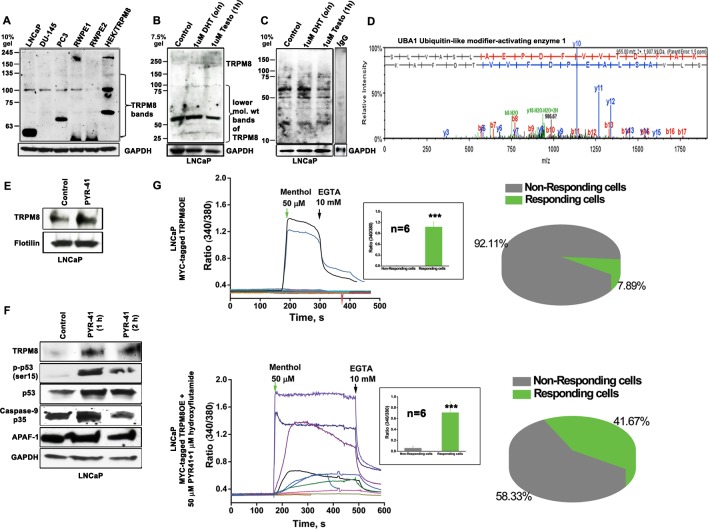
Ubiquitin-mediated proteolytic degradation of TRPM8 in an androgen-responsive LNCaP cells **A.** Immunoblot analysis of cell lysate proteins (160 μg) from 5 PC cell lines and HEK-TRPM8 using anti-TRPM8 antibody. **B.** To determine the lower mol. wt (LMW) product of TRPM8 upon DHT and testosterone-induction, immunoblot analysis of cell lysate proteins (80 μg) was done using anti-TRPM8 antibody. **C.** The control and DHT/testosterone-treated cells were lysed for ubiquitination assays to detect the endogenous ubiquitinated-TRPM8 protein levels. IgG was used as negative control antibody. **D.** The LC-MS/MS spectra analysis of a 130 kDa band, immunoprecipitated from LNCaP cells using anti-TRPM8 antibody identified UBA1. **E.** The cell surfaces of control and PYR-41-treated cells were biotinylated. The eluted proteins were immunoblotted against anti-TRPM8 antibody; flotilin used as loading control. **F.** Western blot analysis of cell lysates proteins (40 μg) **G.** Calcium imaging performed on LNCaP cells, transiently expressing TRPM8 (1.5 μg) and GFP (0.4 μg) constructs. The recordings represent the menthol-induced activation of TRPM8 channels (*n* = 6) in control and drug-treated cells. The summaries of menthol-induced recordings in responding and non-responding cells are represented in a pie chart.

Although the TRPM8 mRNA expression was shown to be elevated in LNCaP cells [10], no channel activation was observed in Ca^2+^ imaging experiments (Figure 2). Therefore, we investigated TRPM8 levels in three different PC (LNCaP, DU145 and PC3) and two prostate epithelial cell lines (RWPE1 and RWPE2), along with HEK-TRPM8, using immunoblot analysis (Figure 4A). All the cell lines showed TRPM8 protein band of predicted size 130 kDa. Surprisingly, we observed additional two lower molecular weight (LMW) bands migrating in the range of 100-55 kDa that were recognized by anti-TRPM8 antibody (Figure 4A). The LMW 55 kDa band was seen more prominently in the androgen-responsive LNCaP cells. Furthermore, the expression of LMW 55 kDa band was decreased in 1 μM testosterone-induced LNCaP cells when compared to control and DHT-induced cells (Figure 4B and Supplementary Figure 2B), indicating differential role of androgens in TRPM8 protein stability.

In order to delineate whether the low TRPM8 activity in LNCaP cells could be mediated by TRPM8 degradation, we performed ubiquitination and mass spectrometry analysis. To test for the possible ubiquitination of TRPM8, we used UbiQapture™-Q Kit, which showed increased capture of TRPM8 protein with anti-ubiquitin antibody in control and DHT-induced cells when compared to testosterone-induced LNCaP cells (Figure 4C). These results indicated increased degradation of TRPM8 in PC cells and suggested that testosterone has a functional role in the stabilization of TRPM8.

Mass-spectrometry analysis of a prominent 130 kDa protein band (corresponding to TRPM8 monomers), obtained by immunoprecipitating TRPM8 from membrane and cytoplasmic extracts of LNCaP cells indicated the presence of UBA1, an initial enzyme in the ubiquitin-mediated degradation (Figure 4D). We hypothesized that UBA1 decreases membrane localization of TRPM8 in LNCaP cells. To explore this, we treated LNCaP cells with PYR-41, a potent UBA1 inhibitor [30]. The addition of PYR-41 (50 μM) significantly (*P < 0.01*) rescued the TRPM8 protein expression in LNCaP cells (Figure 4E and 4F). To ascertain whether cell surface TRPM8 is regulated by UBA1-mediated protein degradation mechanism, we performed biotinylation experiments. PM proteins were labeled with impermeable EZ-Link Sulfo-NHS-SS-Biotin at 4 °C. Equal biotinylated aliquots from control and PYR-41-treated cells were analyzed by western blotting. We observed increased precipitation of TRPM8 protein in PYR-41-treated cells when compared with control LNCaP cells (Figure 4E). These results demonstrated that by targeting the initial enzyme in the ubiquitination cascade, UBA1 by PYR-41 treatment, increases TRPM8 localization on the PM.

Previously it was shown that PYR-41 treatment increases expression and activity of p53, which indicated the ability of PYR-41 to induce apoptosis in transformed cells expressing wild-type p53 [30]. To determine whether PYR-41 treatment in LNCaP cells, with higher TRPM8 activity, can affect the cell viability, we performed immunoblotting to detect the expression of cell regulatory and apoptosis-related molecules. In accordance with the earlier report [30], we also observed enhanced levels of cell cycle inhibitor (p-p53, p53) in PYR-41-treated cells when compared to control cells (Figure 4F). Most interestingly, we observed enhanced levels of cleaved 35 kDa fragment of Caspase-9, which suggests the activation of caspase-9 in the PYR-41 treatment (1 h). However, the major effector caspase, Caspase-3 did not increase (Supplementary Figure 2C) with increased activation of Caspase-9 and the apoptosome-associated molecule, Apaf-1 in PYR-41 (1 h)-treated conditions. These results suggest that the caspase-3 activation requires constant caspase-9 activity throughout the incubation period (Figure 4F). These observations indicate that activation of TRPM8 on the PM can induce p53-depenedent-Caspase-9- mechanism.

### PYR-41/HF treatment enhances _PM_TRPM8 activity

**Figure 5:**
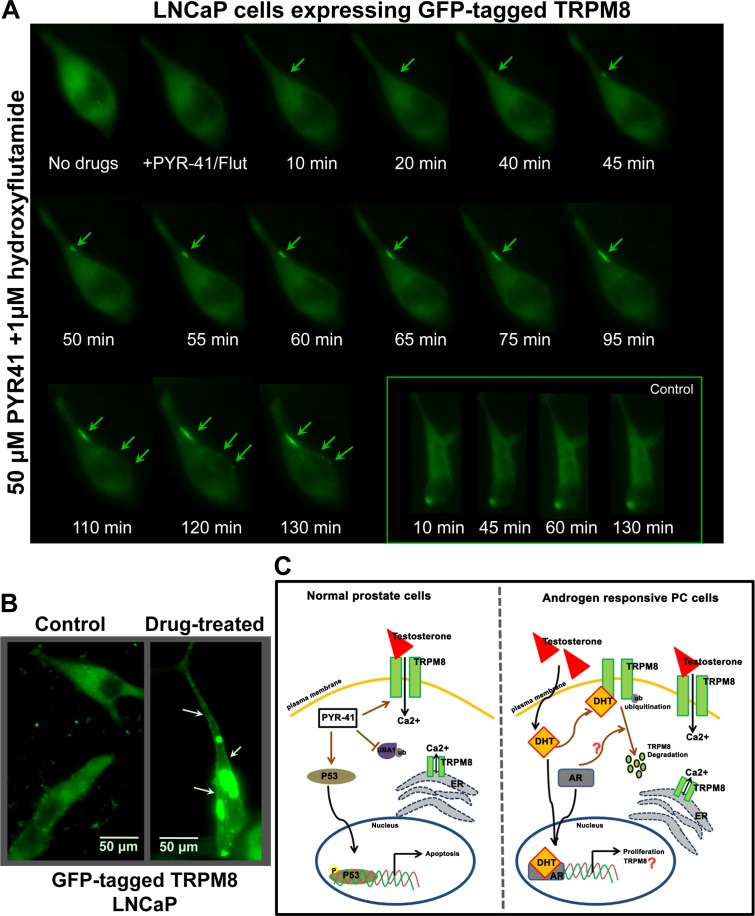
Live-cell imaging for distribution of GFP-TRPM8 **A.** LNCaP cells were transiently transfected with GFP-tagged TRPM8 construct (1.5 μg) and incubated for 48 h. Cells treated with 50 μM PYR-41/1 μM HF and the distribution of the GFP fused TRPM8 was observed at different times (0 to 130 min) after the drug treatment. The live recording was observed using Zeiss-AXIO Observed D1 microscope (Birmingham, NJ). **B.** The confocal microscope (Olympus BX61 Fluoview, Minneapolis, MN) images of LNCaP cells show GFP-TRPM8 translocated to the plasma membrane (indicated by arrows) after the drug treatment. The scale bar represents 50 μm. **C.** Schematic view of TRPM8 regulation in normal prostate and androgen responsive PC progression. Androgens play an important role in the development and progression of PC. The biological responses of androgens are mediated through its receptor, AR. The AR regulates the expression of androgen-responsive genes including prostate specific antigen (PSA) and TRPM8. However, we show that the TRPM8 overexpression in androgen-responsive cells is associated with increased TRPM8 ubiquitination. Furthermore, androgens regulate the TRPM8 protein expression and function, and AR down-regulates its activity. In case of normal prostate cells the activation of tumor suppressor protein, p53 diminishes the androgenic response. Our results demonstrate a negative feedback loop between AR and p53 activation. In addition, the promoter region of *trpm8* possesses a consensus p53 binding site that implies TRPM8 may serve as a downstream target of tumor-suppressor genes. Here we demonstrate that testosterone-induced TRPM8 activity on the plasma membrane controls cell cycle, proliferation, and apoptosis.

Next, we verified TRPM8 activity after the combination PYR-41/HF drug-treatment using Ca^2+^ imaging experiments. TRPM8OE LNCaP cells were treated with PYR-41 (50 μM) along with AR inhibitor, HF (1 μM) for 1 h. The combination drug treatment elicited the TRPM8 response in 42 % of the transfected cells, whereas only 8 % of the transfected cells responded under the untreated conditions (Figure 4G). These results indicate that the substantial increase in Ca^2+^ uptake in PYR-41/HF-treated cells is caused by the enhanced _PM_TRPM8 activity. Further, we confirmed the above data by performing live cell imaging on LNCaP cells transiently expressing GFP-tagged TRPM8 protein alone or in combination with drugs. We observed that the combination drug treatment significantly increases the translocation of TRPM8 to the PM (Figure 5A and Figure 5B).

### Drug-mediated _PM_TRPM8 activity induces apoptotic cell death in PC cells

In addition, we examined the effect of PYR-41 on cell apoptosis and cell cycle distribution by flow cytometry in LNCaP cells. Treatment of cells with 50 μM PYR-41 showed increased proportion of apoptotic cells, as reflected by the increase in sub-G1 peaks (Supplementary Figure 2D). However, TRPM8 knockdown using shRNA (shTRPM8) alone and in combination with PYR-41 treatment showed no detectable apoptosis (Supplementary Figure 2D). These data suggest that PYR-41-induced TRPM8 stabilization has a cytotoxic impact on LNCaP cells.

**Figure 6:**
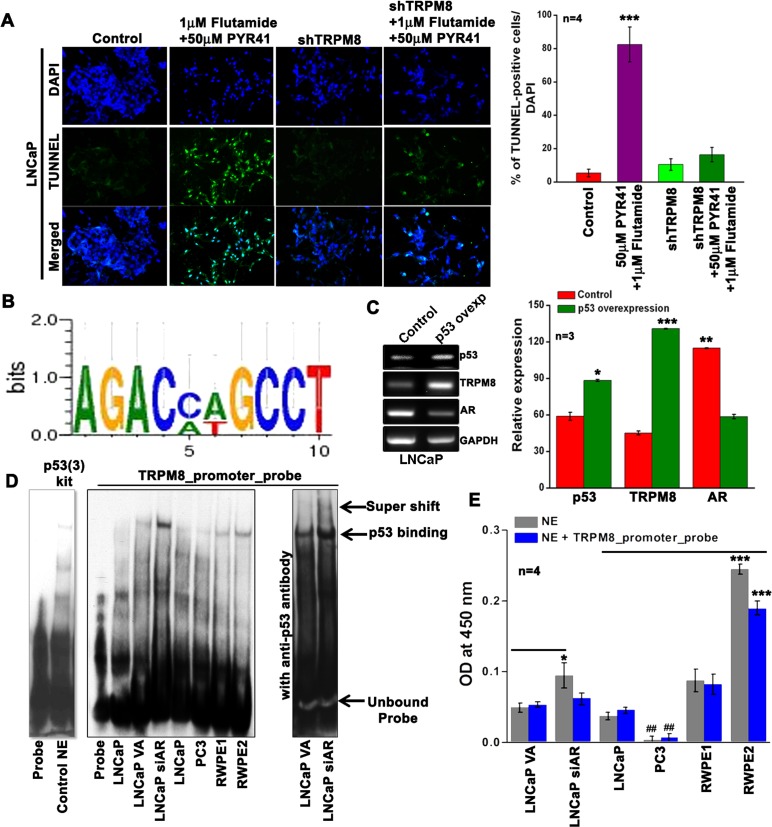
Inverse regulation of TRPM8 by AR and p53 **A.** TUNEL staining on LNCaP cells. Green fluorescence represents apoptotic cells and Blue fluorescence represents DAPI. **B.** The 2064 bp 5′-flanking region of the human *trpm8* gene with ARE I and ARE II sites were analyzed further for the presence of possible putative p53 binding sites using TRANSFAC^®^ 7.0. The authenticity of the TFBS was confirmed by other promoter binding software tools; TFBIND, AlGGEN server PROMO (Version 3.0) and Transcription Element Search Software (TESS). The WebLogo 3.4 software was used to generate the sequence logo for p53 binding site (1399 bp upstream of the transcription start site) on *trpm8* gene promoter. The error bars indicate an approximate, Bayesian 95% confidence interval. **C.** Reverse-transcription PCR analysis of control and p53 overexpressing LNCaP cells (primers are listed in Stable 2). **D.** EMSA was performed with 2 μg of nuclear extract (NE) from control and treated cells. Panel; 1 is for the p53 (3) EMSA probe set in the absence and presence of control NE provided in the kit. Panel; 2 The authenticity of the p53 binding site identified on the TRPM8 promoter was confirmed by performing EMSA using biotinylated probes (STable 3). Panel; 3 shows TRPM8 promoter probe EMSA in the presence of anti-p53 antibody. **E.** Monitoring p53 activation in the NE using TransAM, assay Kit (Active Motif) in the presence and absence of competing TRPM8 promoter probe. The results are graphically represented as bar diagrams.

Further, to elucidate the mechanisms of combined treatment-induced growth inhibition, we examined the effect of PYR-41 and HF on cell apoptosis by staining the cells with TUNEL. We found that the PYR-41/HF-treatment effectively resulted in increased TUNEL-positive LNCaP cells, but it was significantly lower in shTRPM8-transfected cells, indicating that TRPM8 is important mediator of the drug-induced apoptotic cell death (Figure 6A). Thus, the enhanced _PM_TRPM8 may act to increase the Ca^2+^-influx, eventually triggering the cells to undergo apoptosis.

### Trpm8 gene is a downstream target of tumor-suppressor protein p53

Based on the observation that the PYR-41 promotes the transcriptional activity of p53, we investigated whether p53 binding was also localized on the *trpm8* gene promoter. Most interestingly, we found a putative p53-binding site [31] that lies between the positions of ARE I and ARE II (Figure 1B) in the promoter region of human *trpm8* gene (Figure 6B). Further, the reverse-transcription PCR results demonstrated that overexpression of p53 was associated with a significant increase in TRPM8 mRNA levels compared with control LNCaP cells. Reverse-transcription PCR analysis also revealed that overexpression of p53 results in down-regulation of AR mRNA expression levels (Figure 6C), which is consistent with previous studies that demonstrated one level at which p53 affects androgen signaling is through the regulation of AR expression or stability [32].

Further, in order to investigate whether *trpm8* is a direct target of p53-dependent transactivation, we performed EMSA using synthesized double-stranded biotinylated oligos identified by *trpm8* promoter analysis (Figure 6D). We observed that while the nuclear extracts (NE) of LNCaP, PC3, RWPE1, and RWPE2 cells showed p53-binding activity, it was efficiently increased in siAR-transfected LNCaP cells when compared to vector alone (VA)-transfected LNCaP cells. Furthermore, incubation of NE obtained from siAR cells with anti-p53 antibody induced a supershift in p53-*trpm8* promoter probe complex migration (Figure 6D). In addition, we used the TransAM p53 kit to determine the levels of activated p53 in NE of these cells. From the TransAM p53 assay results we observed that in LNCaP, RWPE1 and RWPE2, p53 protein binds to an immobilized oligonucleotide probe containing the p53 consensus sequence. Further, the binding efficiency to this probe was increased in LNCaP cells with knockdown of AR (Figure 6E). These results demonstrate a negative feedback loop between AR and p53 activation. Indeed, the p53 bound to its consensus sequence in TransAM p53 assay was efficiently competed out by the TRPM8 promoter probe (blue bars) (Figure 6E), indicating that p53 interacts specifically with these sites in the *trpm8* promoter.

### Association of endogenous androgens and AR with TRPM8 in subsets of malignant versus normal human prostate tissues

**Figure 7:**
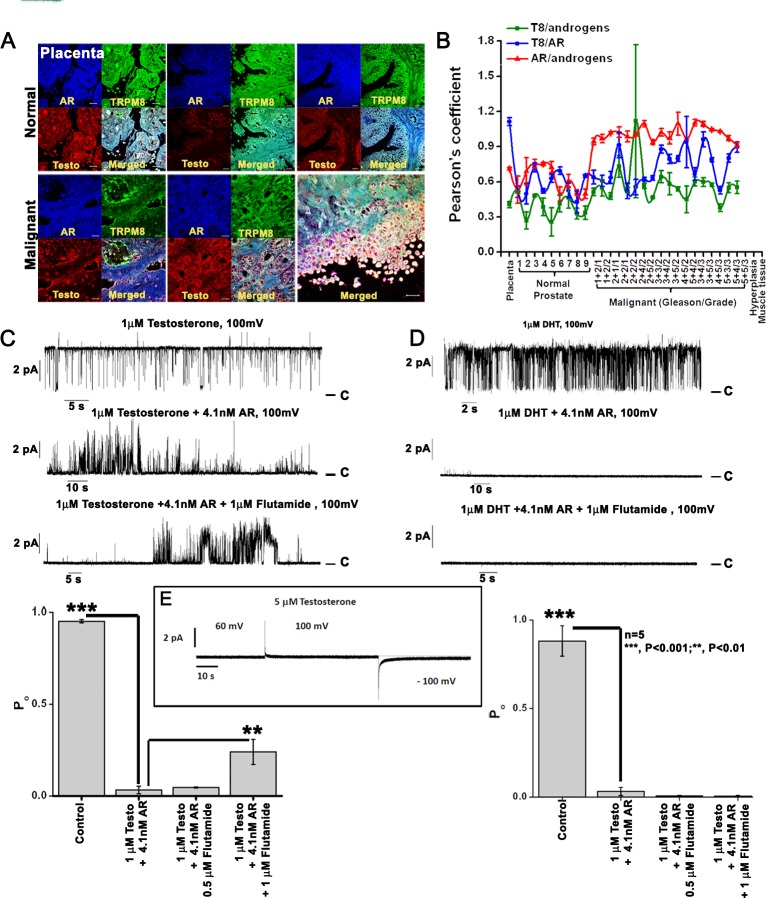
TRPM8 protein is physically associated with androgen-AR proteins **A.** Immunohistochemistry analysis of PC tissue microarray (US Biomax) using Alexa fluor secondary antibodies. Images are at 40X magnification. **B.** The Pearson's coefficient was calculated in ImageJ with the colocalization finder plugin and plotted to show relative co-localization pattern between TRPM8/androgen/AR proteins (*n* = 3). **C-D.** Testosterone and DHT-induced activity of the TRPM8 channel in the planar lipid bilayers. Representative single-channel current recordings of TRPM8 channel openings induced by **C.** testosterone and **D.** DHT application. Addition of purified AR protein inhibited the TRPM8 channel activity. Whereas, addition of HF, an anti-androgen drug that competes with testosterone and DHT for binding to AR showed re-opening of the channel in testosterone-induced but not DHT-induced conditions. Open probability of the TRPM8 channel, obtained at +100 mV was represented graphically. **E.** No current traces obtained up to 5 μM testosterone concentration used as control.

To probe the involvement of androgens and AR in mediating TRPM8 expression in PC, we performed immunohistochemistry analysis on PC tissue microarray containing 60 specimens of PC and 9 specimens of normal prostate tissue, triplicate cores per case (208 cores). We observed that when compared to normal prostate controls the levels of androgens, AR and TRPM8 were abundant in the membrane and cytoplasm of PC tissues. In accordance with the earlier report [33] we observed a shift toward the higher colocalization values for androgen-AR in PC tissues, indicating the enhanced activation of AR [34, 35]. Interestingly, colocalization of TRPM8 with both androgens and AR was also observed in all the tissues. However, the androgen-TRPM8 colocalization remained static in the malignant tissues in comparison to controls, indicating that their interactions do not contribute to the cancer progression. Whereas, TRPM8-AR colocalization fluctuated between the counterpart associations (Figure 7A and 7B).

### Functional role of androgens/AR in TRPM8 ion channel activity in planar lipid bilayers

Recently we demonstrated that testosterone is a highly potent agonist of TRPM8 channels (25). To further determine how AR protein affects TRPM8-testosterone interactions, we tested their functional regulation in planar lipid bilayers. The TRPM8 single channel recordings were obtained within few minutes after addition of testosterone or DHT to the *cis* compartment (Figure 7C and 7D). Importantly, subsequent addition of the purified AR protein resulted in prompt channel inhibition as evidenced by the reduction in open probability of the channel. This inhibition was partial when TRPM8 was activated with testosterone and almost complete when the channel was activated with DHT (Figure 7C and 7D). Further application of AR inhibitor, HF resulted in partial recovery of open probability of TRPM8 activated with testosterone, but not with DHT. This result could be explained by the fact that when compared to testosterone, DHT has nearly a 10-fold higher AR binding affinity [36]. On the other hand, testosterone has higher affinity to TRPM8 (EC_50_ = 65 pM) in comparison to DHT (EC_50_ = 21 nM) [19]. These results indicate that an inhibitory effect exerted by AR on androgen-induced TRPM8 activity is due to the competitive binding.

Together our results indicate that the prostate tumor growth and development is associated with a complex androgen-TRPM8-AR regulatory loop.

## DISCUSSION

TRPM8 is a Ca^2+^ permeable, non-selective cation channel of the transient receptor potential superfamily. Ca^2+^ signaling regulates the proliferation and apoptotic pathways in cancer cells [37] and therefore the altered expression or activity of Ca^2+^ channels can substantially affect Ca^2+^ homeostasis.

Human TRPM8 was initially identified and cloned as a prostate-specific gene in PC cells [10]. More precisely, TRPM8 mRNA is overexpressed in well-differentiated early prostate tumors with high androgen levels, while anti-androgen therapy greatly reduces the expression of the protein [11]. Although many hypotheses have been put forward, the prostate-specific function of Ca^2+^-permeable channel TRPM8 in prostate physiology and carcinogenesis remain unknown. LNCaP cells, a typical model of androgen-dependent PC shows TRPM8 localization in the endoplasmic reticulum (ER) membranes, where it supports ligand-induced Ca^2+^ release [20, 38]. However, _PM_TRPM8 is not functional in LNCaP cells. We identified that the high TRPM8 mRNA expression levels [10] compared to lower _PM_TRPM8 protein abundance and activation in LNCaP cells is associated with increased TRPM8 ubiquitination. The _PM_TRPM8 might exert a protective effect, since functional activation of _PM_TRPM8 by prostate specific antigen (PSA) reduced the cell motility in PC3 cells [39]. In our studies, we observed that PYR-41, a potent inhibitor of initial enzyme in the ubiquitination cascade (UBA1), increased TRPM8 activity on the PM of LNCaP cells. Further, PYR-41-mediated _PM_TRPM8 activity was accompanied by enhanced activation of p53 and Caspase-9. In addition, the promoter region of *trpm8* possesses a consensus p53 binding site that implies TRPM8 may serve as a downstream target of tumor-suppressor genes.

Our data indicate that _PM_TRPM8 plays a protective role in PC progression. Previously, in PC3 cells with low levels of functional TRPM8, overexpression of TRPM8 induced anti-proliferation and pro-apoptotic effects. The cell cycle arrest and reduced cell motility was through down-regulation of Cdk4/6 and FAK respectively [40]. Further, both TRPM8 overexpression and menthol treatment showed increased cytoplasmic Ca^2+^ levels in PC3 cells [40]. Interestingly, we found that treatment of LNCaP and PC3 cells with TRPM8 agonist, menthol accompanied by AR inhibition or TRPM8 overexpression, respectively, showed greater anti-proliferative effect. Furthermore, the PYR-41/HF combination drug treatment significantly increased the susceptibility of LNCaP cells to apoptosis accompanied by the increased _PM_TRPM8 activity.

The extremely high levels of AR in LNCaP cells contribute to high levels of TRPM8 mRNA expression probably due to higher response to androgen regulation [21]. Although the AR activation is shown to be a key element in DHT-mediated up-regulation of *trpm8* gene expression [20], still no evidence was shown for direct regulation of the *trpm8* gene by AR in the prostate. We identified that the promoter region of *trpm8* possesses functional ARE I binding site with transcriptional regulatory function. However, the ChIP data demonstrated inverse correlation of androgen-mediated *trpm8* promoter regulation with androgen response of PC cells. All of these studies and our observations indicate that TRPM8 protein could be positively regulated at the transcriptional level and negatively regulated at the post-translational level by AR.

Several studies support the idea that the TRPM8 mRNA expression in PC can be used as a prognostic marker and as a potential therapeutic target [10, 21, 41, 42]. When compared to various potential PC-associated antigens, TRPM8 mRNA expression was confined to prostate organ [12, 43] and showed no strong correlation of expression patterns between tumorigenic and normal prostate tissues [13]. Of five selected TRPM8-derived peptides, only peptide GLMKYIGEV was shown to activate specific cytotoxic T lymphocytes responses *in vitro*, which failed to induce an effective increase of TRPM8-reactive CD8þ T-lymphocytes in human patient vaccination studies [44]. All these data provide evidence that in early stage of human PC development, the normal immunological defense mechanism may enhance the destruction of PC cells expressing TRPM8. A growing body of evidence suggests that unleashing the androgen-independent effects [39, 45, 46] can clarify the possibility that TRPM8 expression in PC may act as a negative regulator in the relevant context.

As the *trpm8* gene was silenced in PC tissue from patients treated preoperatively with anti-androgen therapy, Henshall *et al.* suggested that the *trpm8* gene was under androgen control [11]. The studies showed that the loss of TRPM8 mRNA expression was associated with a significantly shorter time to PSA relapse-free survival and that the patient with lower *trpm8* expression would have a 4-fold increased risk of relapse [11]. Despite a large body of literature addressing the expression and function of TRPM8 in prostate, its role has remained elusive since its discovery. This was mainly due to the unknown endogenous agonists of this channel, as well as its physiological and pathological functions in PC. Recently we identified that TRPM8 is as an ionotropic testosterone receptor [47], which determines its physiological role in prostate.

Thus, androgens, which are at the crossroads of several signaling pathways, appear to be associated with TRPM8-mediated Ca^2+^ signaling. In our recent studies [18, 19], we reported for the first time that androgens are both physically and functionally associated with TRPM8 protein. The current study extended our previous findings on androgen-TRPM8 interactions, corroborating the negative regulation of PC cell growth and proliferation. Most strikingly, we found that the androgen-mediated TRPM8 channel activity is negatively regulated by AR protein. Our results on the functional activation and ubiquitination of TRPM8 may support a strategy for targeting the androgen-TRPM8-AR interaction or rescuing _PM_TRPM8 expression as a new therapeutic application to treat PC patients in the future (Figure 5C).

## MATERIALS AND METHODS

### Ethics statement

Human high-density PC tissue microarray was purchased from US Biomax (Rockville, MD). All the prostate tissue samples were obtained from patients (Age: 21 yrs to 83 yrs) undergoing therapeutic surgery at the US Biomax certified hospitals (see Sdata).

### Cell lines, transfection

The cell lines RWPE1, RWPE2, LNCaP, DU145 and PC3 were obtained from the American Type Culture Collection (Manassas, VA) and were maintained under standard conditions as per ATCC guidelines. LNCaP, PC3 and DU145 cells were cultured in RPMI, F-12K and DMEM/F12K (1:1) medium, respectively supplemented with 10% fetal bovine serum (GIBCO BRL, Lewisville, TX) and 1% penicillin/streptomycin. RWPE1 and RWPE2 cells were grown in keratinocyte-serum free media (K-SFM) containing 50 μg/ml bovine pituitary extract, 5 ng/ml epidermal growth factor and 1% penicillin/streptomycin solution. All cells were cultured in a 37°C incubator with 5% CO_2_ humidified atmosphere. HEK-293 stable cells overexpressing TRPM8 (HEK-TRPM8) were established and maintained following standard protocol [48]. All transfection experiments were performed using FuGENE HD transfection reagent (Roche Diagnostics, Indianapolis, IN) (see Sdata).

### Plasmids, shRNA construct, antibodies and chemical inhibitors

TRPM8 rat cDNA cloned in N-terminus myc-tagged pCDNA3 vector for TRPM8 overexpression (TRPM8OE). TRPM8 trafficking investigated by GFP-TRPM8. The siAR and shTRPM8 purchased from Santa Cruz Biotechnology (Santa Cruz, CA). pCDNA3-empty vector used as control. All antibodies purchased from Santa Cruz Biotechnology; anti-TRPM8/CMR-1 (656-680) from Phoenix Pharmaceuticals (Burlingame, CA); anti- DHT/testosterone from Thermo Scientific Pierce (Rockford, IL); Alexa Flour 488 and 594 purchased from Invitrogen (Invitrogen, Carlsbad, CA). Testosterone, DHT (5α-dihydrotestosterone), PYR-41 (UBA1 inhibitor) and hydroxyflutamide (HF; AR inhibitor) was purchased from Sigma (St. Louis, MO).

### Chromatin immunoprecipitation (ChIP) and real time-PCR (RT-PCR)

ChIP assay was performed using ChIP-IT^TM^ Express kit from Active Motif (Carlsbad, CA) following supplied protocol. (see SMethods).

### Sub-cellular protein fractionation, immunoprecipitation, immunoblotting and mass spectrometry

Detergent soluble membrane fraction preparation and analysis was done according to standard protocol [25, 49].

### Ubiquitination and biotinylation assay

For the ubiquitination assay, whole cell lysates (500 μg) were incubated with UbiCapture-Q Matrix (VWR International, Batavia, IL). Biotinylation assays were performed using cell surface protein isolation kit (Thermo Scientific Pierce) (see SMethods).

### Transmission electron microscopic studies

The cells were fixed using fixative solution (2.5% glutaraldehyde in 0.1 M phosphate buffer, pH 7.4). After fixation, samples were buffer rinsed and postfixed with 1% osmium tetroxide, dehydrated (35%, 70%, 95%, 100% ethanol dehydration), and flat embedded in propylene oxide and Epon 812 epoxy resin (Tousimis) in 1:1 ratio at 60 °C for 4 h. A Reichert OMU3 ultramicrotome (Austria) was used to prepare 700A° thin sections that were mounted on 200 copper mesh grids, stained with uranyl acetate and lead citrate. The sections were viewed under a JEOL JEM 100C transmission electron microscope (60 kV).

### FACS, TUNEL and clonogenic assay

The effects of androgen / AR inhibition / TRPM8 overexpression on cancer cell proliferation were examined by FACS, TUNEL and clonogenic assay following standard protocol [47] (See SMethods).

### EMSA and p53 transcription factor assay

The p53 activation in nuclear extracts was monitored using p53 (3) - EMSA (Affymetrix, Santa Clara, CA) and ELISA-based TransAM™ p53 kit (Active Motif) according to the manufacturer's protocol (see SMethods).

### Calcium imaging and planar lipid bilayers

Fluorescence measurements of intracellular Ca^2+^ concentration of single cells were measured using standard protocol [19, 25, 48]. Planar lipid bilayers measurement and purification of TRPM8 protein from HEK-TRPM8 cells done using standard protocol [50].

## SUPPLEMENTARY MATERIAL FIGURES AND TABLES


