# Supplementary material for: TRPM8 channel as a novel molecular target in androgen-regulated prostate cancer cells
Source: Oncotarget. 2015 Apr 29;6(19):17221–36. doi: 10.18632/oncotarget.3948 (PMC4627303; doi:10.18632/oncotarget.3948)
Supplement: Supplementary file 1 [file oncotarget-06-17221-s001.pdf]

TRPM8 channel as a novel molecular target in androgen-regulated prostate cancer cells

Supplementary Material

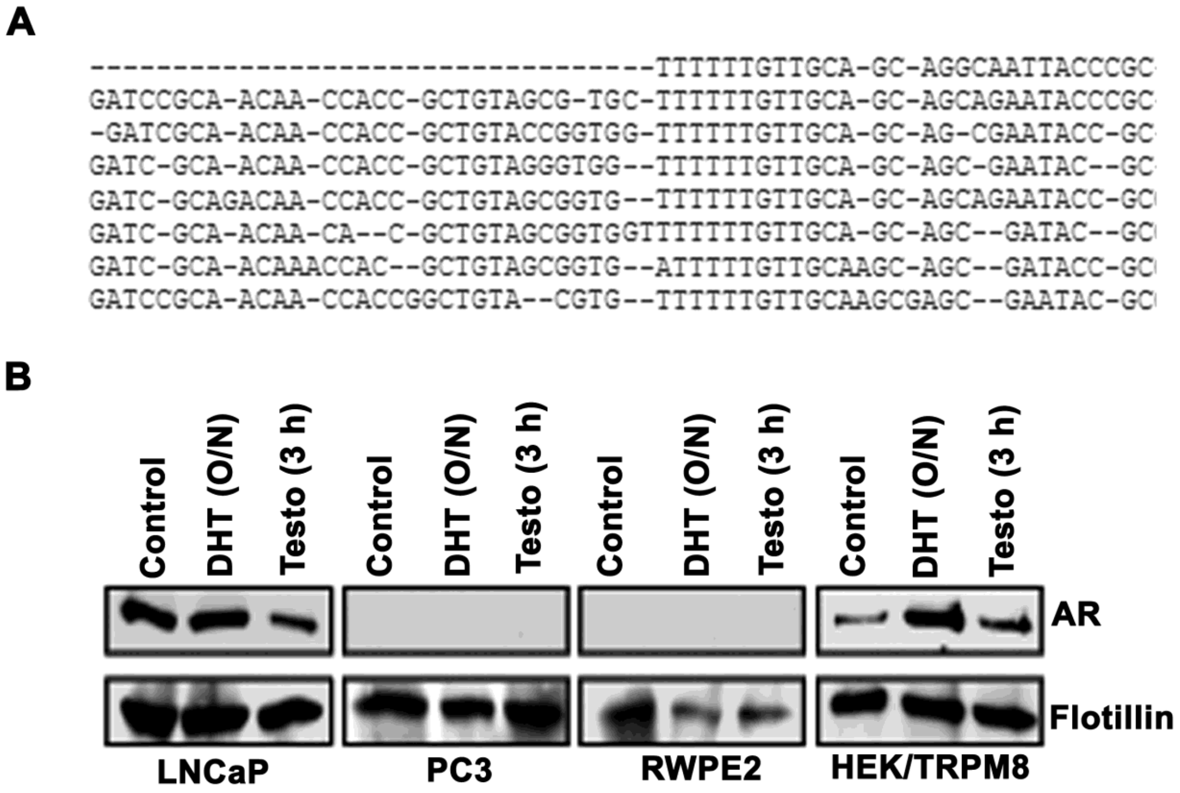

Supplementary Figure. 1: (A) The representative clones of Chromatin immunoprecipitation (ChIP) using anti-DHT/testosterone antibody, performed in LNCaP control and DHT-induced (o/n) cells. The ChIP eluted chromatin was subjected for cloning and sequencing analysis as described in Materials and Methods. The sequencing data from the clones were filtered using *VecScreen* and *NCBI blast tool* and aligned using ClustalW2 multiple sequence alignment program (http://www.ebi.ac.uk/Tools/msa/clustalw2/). **(B)** Western blot analysis of detergent solubilized membrane fractions to demonstrate the levels of androgen receptor (AR); flotillin was used as a loading control.

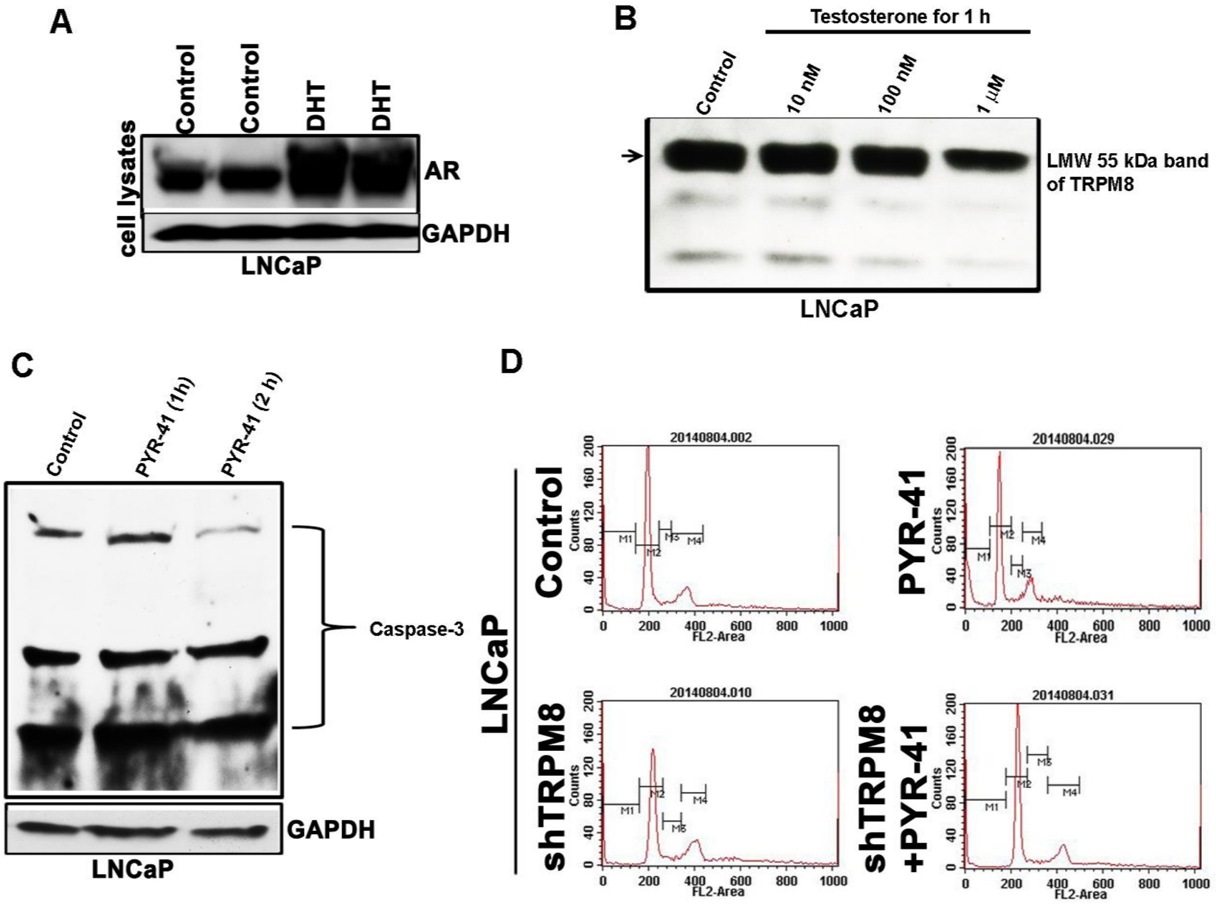

Supplementary Figure. 2: **(A)** Western blot analysis of cell lysates to demonstrate the levels of androgen receptor (AR); GAPDH was used as a loading control. **(B)** To determine the levels of lower mol. wt (LMW) product of TRPM8 with increasing concentrations of testosterone-induction in LNCaP cells, immunoblot analysis of cell lysate proteins (80 µg) was done using antibodies specific for TRPM8. **(C)** Western blot analysis of cell lysates to demonstrate the levels of cleaved Caspase-3**.** **(D)** The cell cycle progression in LNCaP control and shRNA specific for TRPM8 (shTRPM8) transfected cells alone and in combination with 50 μM PYR-41 treated cells.

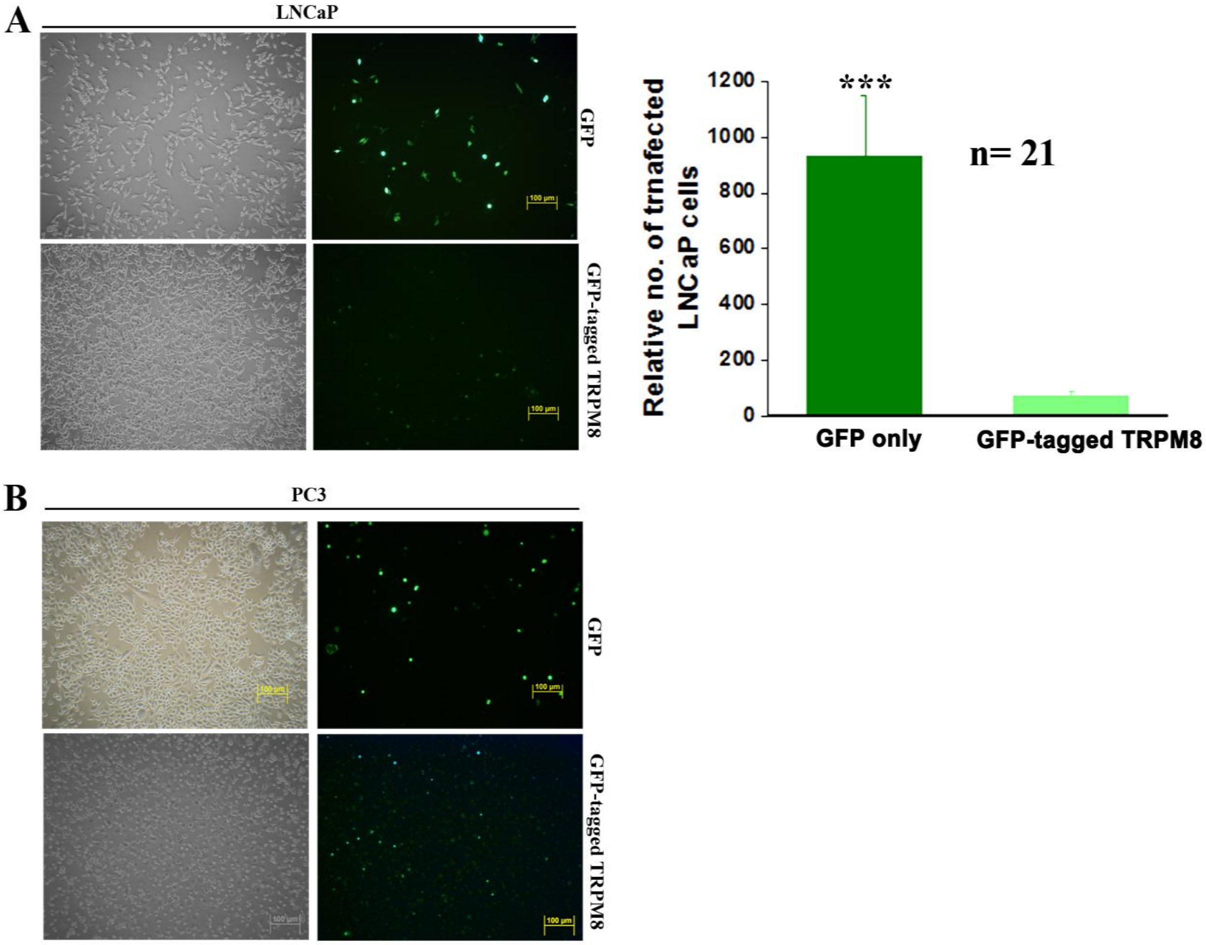

Supplementary Figure. 3: The figure shows transfection efficiency of GFP (0.4 μg) and GFP-tagged TRPM8 (1.5 μg) constructs in **(A)** LNCaP cells and **(B)** PC3 cells. The transfection of only GFP constructs did not vary in LNCaP and PC3 cells. However, we observed that the transfection efficiency of GFP-tagged TRPM8 in PC3 cells was higher than in LNCaP. PC3 cells showed massive cell death after 48 h of transfection with GFP-TRPM8.

Supplementary Materials and Methods

Transfection

All transfection experiments were performed with FuGene *HD* transfection reagent (Roche, Indianapolis, IN). Briefly, plasmid was mixed with FuGene *HD* reagent (1:3 ratio) in 200-500 µl of serum free-medium and left for 30 min at RT. The complex was then added to the 6-well or 100 mm plate containing 0.5 mL or 3 mL of serum-free medium, and around 1 μg plasmid per mL of medium was used. After 8 h of transfection, complete medium containing 10 % FBS was added, and incubated for 64 h. Control cells were processed in the same way as treated cells and were incubated with either equal volumes of FuGENE HD or transfected with vector alone.

Immunocytochemistry and immunohistochemistry

For immunocytochemical analysis, treated cells were seeded onto 4-well chamber slides, fixed, permeabilized with ice-cold methanol and blocked for 1 h using 3% BSA in PBS. Cells were incubated with primary antibody and Alexa Fluor conjugated secondary antibody for 60 min at room temperature. Before mounting, the slides were washed with PBS and incubated for 5 min with a 1:100 dilution of 4'-6-Diamidino-2-phenylindole (DAPI) for nuclear staining. For immunohistochemical analysis, prostate tissue array (PR208) sections were deparaffinized in xylene, rehydrated in graded ethanol solutions. Antigen retrieval was carried out with 10 mM citrate buffer (pH 6) at boiling temperature for 60 min and permeabilization in 0.1% Triton-X-100. The tissue sections were incubated with primary antibodies for DHT/Testosterone, TRPM8 and androgen receptor (AR) for overnight, incubated with donkey Alexa Fluor conjugated secondary antibodies. The images were captured using confocal microscopy (Olympus BX61 Fluoview, Minneapolis, MN) at 40X magnification.

Chromatin immunoprecipitation (ChIP) assay

Chromatin was sheared by sonication method. The anti-DHT/testosterone or control IgG antibody was used to immunoprecipitate the chromatin processed from LNCaP, PC3 and HEK/TRPM8 control and testosterone-induced cells for overnight at 4^0C followed by DNA purification and elution. The ChIP-enriched DNA was subjected to end filling using End-It™ DNA End-Repair Kit (EPICENTRE, Madison, Wisconsin). The end repaired ChIP DNA was cloned into pGEM-T easy Vector System-II (Promega, Madison, WI) containing T7 and SP6 RNA polymerase promoters flanking a multiple cloning region within the alpha-peptide coding region of the enzyme beta-galactosidase. The recombinant clones were screened by blue/white selection. The plasmids were isolated

from the subclones (QIAprep Spin Miniprep Kit, QIAGEN, Maryland) and subjected to the restriction digestion and sequencing analysis (DNA CoreLIMS, Urbana, IL).

For the analysis of DHT/testosterone binding to TRPM8 promoter, the ChIP DNA immunoprecipitated by anti-DHT/testosterone and anti-IgG antibody was used as template for ChIP specific primers which were designed to cover the Regions 1-10 (R1, R2, R3, R4, R5, R6, R8, R9 and R10) of TRPM8 gene promoter (ref|NW_004929306.1) and were used to perform quantitative RT-PCR following standard protocol. The coefficient of androgen/AR interaction with different regions (R1-R10) in the TRPM8 promoter was quantified and represented graphically. The ChIP specific R1-R10 primer sequences are listed in Supplementary table 1.

Supplementary Table 1. The ChIP specific R1-R10 primer sequences

Regions	Forward Sequence	Reverse Sequence
R1	5'- TTGCTAGATGAATTGGGCATT - 3'	5'- ACGTGAATGGATCCTTGCTG -3'
R2	5'- CCATCTCTCTCGCTCCCATT - 3'	5'- AAATCCAAACTTGTTTTATCCACA - 3'
R3	5'- GCTGTGGATAAAACAAGTTTGGA - 3'	5'- GCTGTGGATAAAACAAGTTTGGA - 3'
R4	5'- ACTCTGTCACCCAGGTTGGA - 3'	5'- ACATGGCAAAACCCTGTCTC - 3'
R5	5'- TCCCAAAGTGCTGGGATTAC - 3'	5'- CCCGTGAGAGAAGAGATCCA - 3'
R6	5'- CATGCTGGGTATGGGAGTTT - 3'	5'- AAGGGAGCAGCCAAGTGTTA - 3'
R7	5'- CTCCCAAAGTGCTGGGATTA - 3'	5'- AAGGGAGCAGCCAAGTGTTA - 3'
R8	5'- CTCTTCTCTCACGGGTCCTC - 3'	5'- CCAGACAGAGGTCTCAAACTCA - 3'
R9	5'- ATGGCCAAAACAGAGGAACA - 3'	5'- AGGTGAGCAAATCCAGCTTC - 3'
R10	5'- GGAAAGTTCACAAAATCTGCAA - 3'	5'- AGGATCCTGCTGGTCTCTCC - 3'

Real-Time PCR (RT-PCR) analysis

Total RNA was isolated using TRIZOL reagent (Invitrogen, Carlsbad, CA). 1 μg of RNA was subjected to reverse transcription using the Superscript III reverse transcriptase cDNA synthesis kit (Roche Applied Science, Indianapolis, IN). Reverse transcription reaction was carried out in a thermal cycler for 30 minutes at 55°C followed by 5 minutes at 85°C. Real-time PCR analysis was then performed in triplicate using the SYBR Green PCR Master Mix (Applied Biosystems). The following PCR conditions were used: 95°C for 10 minutes, followed by 40 cycles at 95°C/10 sec and 60°C/30 sec. The fold enrichment of androgen interaction with different regions (R1-R10) in the *trpm8* promoter was quantified and was normalized to ChIP DNA immunoprecipitated by anti-IgG antibodies.

Reverse-transcription PCR.

Total RNA was isolated from control and treated cells using TRIZOL reagent (Invitrogen, Carlsbad, CA) according to the standard protocol. Using 1 μg of RNA as a template, first-strand cDNA was synthesized using Superscript III reverse transcriptase (Roche Applied Science, Indianapolis, IN). PCR analysis using 100 ng of first-strand cDNA was completed using specific primers (Supplementary Table 2). GAPDH was used as an internal control. The PCR reaction was performed using standard conditions in an AB Applied Biosystems Thermocycler (model 9700). PCR amplified products were electrophoresed on 1.5% agarose gel.

Supplementary Table 2. Reverse-transcription PCR primers for the genes

Regions	Forward Sequence	Reverse Sequence
trpm8	5'- CATTGTGTGTTTTGCCCAAG - 3'	5'- TTGACGGCAGAAGATGTCAG -3'
AR	5'- CCAGGGACCATGTTTTGCC - 3'	5'- CGAAGACGACAAGATGGACAA - 3'
p53	5'- ACTTGTCGCTCTTGAAGCTAC - 3'	5'- GATGCGGAGAATCTTTGGAACA - 3'
GAPDH	5'-GTTGCCATCAATGACCCCTT- 3'	5'-CTCCACGACGTACTCAGCG- 3'

LC/MS

LC-MS/MS spectra was generated following standard protocol (see SMethods) at University of Florida. Briefly, the enzymatically digested samples were injected onto a capillary trap (LC Packings PepMap) and de-salted for 5 min with a flow rate of 3 µl / min of 0.1% v/v acetic acid. The samples were loaded onto an LC Packing® C18 Pep Map nanoflow HPLC column. The elution gradient of the HPLC column started at 3% solvent A, 97% solvent B and finished at 60% solvent A, 40% solvent B for 30 min for protein identification. Solvent A consisted of 0.1% v/v acetic acid, 3% v/v ACN, and 96.9% v/v H2O. Solvent B consisted of 0.1% v/v acetic acid, 96.9% v/v ACN, and 3% v/v H2O. LC-MS/MS analysis was carried out on a hybrid quadrupole-TOF mass spectrometer (QSTAR XL, Applied Biosystems, Framingham, MA). The focusing potential and ion spray voltage was set to 225 V and 2400 V, respectively. The information-dependent acquisition (IDA) mode of operation was employed in which a survey scan from m/z 400–1800 was acquired followed by collision-induced dissociation (CID) of the four most intense ions. Survey and MS/MS spectra for each IDA cycle were accumulated for 1 and 3 seconds, respectively. The obtained spectra was analysed by using scaffold (™) software version

3_00_07 (Portland, OR). The cut off for these putative identified proteins were set to 99 % identity.

Ubiquitination assay

For the ubiquitination assay, whole cell lysates (500 μg protein/sample) were incubated with UbiCapture-Q Matrix (VWR International) by gentle agitation at 4°C overnight to pull down all ubiquitinated proteins according to the manufacturer's instructions. After washing three times, captured proteins were eluted with 2× SDS-PAGE loading buffer and analyzed by western blotting using anti–TRPM8 antibody.

Biotinylation assay

Biotinylation assays were performed using Pierce Cell Surface Protein Isolation kit (Thermo Scientific). Three 10 cm^2 dishes of 80%–90% confluent LNCaP cells were harvested. Cells were washed twice with PBS and incubated with 0.25 mg/ml of sulfo-NHS-SS-biotin in PBS for 30 min. After addition of 300 μl of quenching solution, cells were gently scrapped and centrifuged at 1000× g for 3 min, washed with TBS and centrifuged again. Cells were then lysed with 500 μl of lysis buffer (Thermo Scientific) supplemented with 1% PMSF and 1% protease inhibitors (ProteCEASE). After sonication on ice (five 1 s bursts), cells were incubated on shaker for 30 min at 4°C. After centrifugation at 10,000 × g for 2 min at 4°C, supernatants were transferred to a new tube. Neutravidin beads (Pierce) were prepared as described by Thermo Scientific and incubated with supernatant on a rotating shaker for 60 min at RT. After centrifugation at 1000 × g for 1 min, beads were washed three times with 400 μl of wash buffer. Elution was performed with 120 μl of sample buffer containing DTT by heating for 4 min at 95°C. Samples were then processed for Western blot analysis and visualized by using an anti-TRPM8 antibody.

Cell cycle analysis by fluorescence-activated cell sorting (FACS)

The treated cells were harvested by trypsinization and stained with propidium iodide (PI) (2 mg/mL) (Biosure, Grass Valley, CA). Suspensions of $1{\times}10^6$ cells were analyzed by FACS Caliber System (Becton Dickinson Bioscience, San Jose, CA) with laser excitation at 488 nm and emission at 639 nm. The percentages of cells in the various phases of the cell cycle (G0/G1, S, and G2/M) were assessed using Cell Quest software (Becton Dickinson Bioscience, San Jose, CA).

TUNEL assay

The TUNEL assay was done using *in situ* cell death detection kit (Roche) in accordance with the manufacturer's protocol. In brief, control and treated LNCaP cells were fixed in 4-well chambered slides with 4% paraformaldehyde in 0.1 M phosphate buffer (pH 7.4). Next, cells were incubated with a TUNEL reaction mixture for 60 min at 37°C in a humidified incubator. The slides were washed 3 times with phosphate-buffered saline (PBS), and the incorporated biotin-dUTP was detected under a fluorescence microscope (Olympus BX61 Fluoview, Minneapolis, MN) at 40X magnification.

Clonogenic assay

The single cell suspension of LNCaP and PC3 control and treated cells were prepared following Franken *et., al* 2006 protocol. About 500 cells were individually plated in 60 mm plates, spread well and incubated for 10-15 days until cells in control plates had formed sufficiently large colonies. Next, the cells were stained with HEMA-3 for 10 min and colonies were counted and graphically represented.

Intracellular Ca^{2+} Measurements

The extracellular solution used in ratiometric [$\mathbf{Ca^{2+}}$]i measurements contained 137 mM NaCl, 5 mM KCl, 1.8 mM CaCl2, 1 mM MgCl2, 10 mM glucose, and 10mM Hepes, pH 7.4. Cells were incubated with 2 µM Fura-2 acetoxymethyl ester (Tef Labs, Austin, TX) for 30 min at room temperature. The fluorescence signals of the cells grown on the coverslips were measured using alternating excitation at 340 and 380 nm, and emission was detected at 510 nm. The ratio of fluorescence (340 nm/380 nm) was plotted against time. The obtained values of ratios from each coverslips were first analyzed, and then the mean values of stimuli-induced signals were combined, and statistically averaged values with mean errors were plotted in the summary graphs. The total number of measurements/coverslips (n) are indicated in the figure legends. The measurements were performed using a Photon Technology International (Birmingham, NJ) imaging system mounted on an Zeiss-AXIO observed D1 microscope, equipped with a DeltaRAM excitationlight source or with a Ratiomaster 5 imaging system (PhotonTechnology International) equipped with a Cool-snap HQ2(Roper) camera.

Preparation of the TRPM8 Protein from Human Embryonic Kidney (HEK-293) Cells

HEK-293 cells stably expressing TRPM8 were grown to 70–80% confluence, washed, and collected with PBS. Cells were harvested and resuspended in NCB buffer, containing 500 mM

NaCl, 50 mM NaH2PO4, 20 mM Hepes, 10% glycerol, pH 7.5, with the addition of 1mM of protease inhibitor PMSF, 5 mM β-mercaptoethanol. Then the cells were lysed by the freeze-thawing method and centrifuged at low speed to remove cell debris and DNA. The supernatant was further centrifuged at 40,000 x *g* for 2.5 h, and the pellet was resuspended in NCB buffer with the addition of a protease inhibitor mixture (Roche Applied Science), 0.1% Nonidet P-40 (Roche Applied Science), and 0.5% dodecylmaltoside (Calbiochem). The suspension was incubated overnight at 4 °C on a shaker with gentle agitation and then centrifuged for 1 h at 40,000 x *g*. Further, the TRPM8 protein was purified by immunoprecipitation with anti-Myc-IgG conjugated to A/G protein magnetic beads (Thermo Scientific Pierce), following the procedure provided by the manufacturer. All steps of the purification were performed at 4 °C. For the planar lipid bilayer experiments, the protein was eluted with Myc-peptide (50 µg / ml).

Planar Lipid Bilayer Measurements

Planar lipid bilayer measurements were performed as described previously. Planar lipid bilayers were formed from a solution of synthetic 1-palmitoyl-2-oleoyl-glycero-3- posphocoline (POPC) and 1-palmitoyl-2-oleoyl-glycero-3-phosphoethanolamine (POPE; Avanti Polar Lipids, Birmingham, AL) in a 3:1 ratio in *n*-decane (Sigma-Aldrich). The solution was used to paint a bilayer in an aperture of 150 µm diameter in a Delrin cup (Warner Instruments, Hamden, CT) between symmetric aqueous bathing solutions of 150 mM KCl, 0.2 mM MgCl2, 1 µM CaCl2, 20 mM Hepes, pH 7.4, at 22 °C. All lipid bilayer experiments were performed in the presence of phosphatidylinositol 4,5-bisphosphate (PIP2), unless specifically omitted as indicated. 2.5 µM DiC8-PIP2 (Cayman Chemical Comp.) dissolved in water was added to both compartments. All salts were ultrapure (>99%) (Sigma). Bilayer capacitances were in the range of 50–75 picofarads. After the bilayers had been formed, the TRPM8 protein from the micellar suspension (20 ng/ml) was added by painting. Unitary currents were recorded with an integrating patch clamp amplifier (Axopatch 200B, Axon Instruments). The trans solution (voltage command side) was connected to the CV 201A head stage input, and the cis solution was held at virtual ground via a pair of matched Ag-AgCl electrodes. Currents through the voltage-clamped bilayers (background conductance _1 pS) were filtered at the amplifier output (low pass, -3 decibels at 10 kHz, 8-pole Bessel response). Data were secondarily filtered at 100 Hz through an 8-pole Bessel filter (950 TAF, Frequency Devices) and digitized at 1 kHz using an analog-to-digital converter (Digidata 1322A, Axon Instruments), controlled by pClamp version 10.3 software (Axon

Instruments). Single-channel conductance events, all points histograms, open probability, and other parameters were identified and analyzed using the Clampfit version 10.3 software (Axon Instruments).

p53 transcription factor assay

The p53 activation in nuclear extracts of PC cells was monitored using the ELISA-based TransAM™ p53 kit (TransAM p53, Active Motif, CA) according to the manufacturer's protocol. 2μg of total nuclear protein were loaded onto a 96-well plate coated with an immobilized oligonucleotide containing a p53 consensus binding site. Anti-p53 and anti-rabbit HRP were used to quantify the amount of bound p53 protein. The HRP signal was developed by a substrate provided by the manufacturer, and samples were analyzed with an ELISA reader at 450 nm. The OD value for negative control was deducted from all the test samples.

Supplementary Table 3. Synthesized double-stranded biotinylated oligos identified on the TRPM8 promoter used in EMSA studies

Regions	Forward Sequence	Reverse Sequence
TRPM8_Promoter_ p53-Bmd	/5Biosg/CCATGTTGCCCAGGCTGGTCTTG AACCCCT	/5Biosg/AGGGGTTCAAGACCAGCCTGGGC AACATGG

Statistical Analysis

Statistical analyses were performed using Origin 8.0 software (Microcal Software Inc., Northampton, MA). Statistical significance was calculated using one-way ANOVA combined with Fisher's LSD test and data were presented as mean ± SEM. Statistical differences are presented at *$*P < 0.05$, $**P < 0.01$ and $***P < 0.001$.*
